# Have Elderly Individuals in Urban China Been Satisfied with Nursing Services during the COVID-19 Pandemic?

**DOI:** 10.3390/ijerph182010624

**Published:** 2021-10-11

**Authors:** Shoujin Shan, Zhonggen Sun, Furong Zhang, Ruilian Zhang, Bingqing Yang

**Affiliations:** 1 School of Marxism, Hohai University, Nanjing 211100, China; sokin@hhu.edu.cn; 2 School of Marxism, Maanshan Teacher’s College, Maanshan 243041, China; 3 School of Public Administration, Hohai University, Nanjing 211100, China; sunzhonggen@hhu.edu.cn (Z.S.); 201314060012@hhu.edu.cn (F.Z.); yangbingqing@hhu.edu.cn (B.Y.); 4 Sustainable Minerals Institute, The University of Queensland, Brisbane 4067, Australia

**Keywords:** customer satisfaction index model, elderly satisfaction, nursing services, COVID-19, China

## Abstract

Based on the customer satisfaction index model, we constructed an elderly care service satisfaction model that includes 5 latent variables and 16 observed variables. To analyze the degree of satisfaction of elderly individuals in nursing homes, we used structural equation model (SEM) to test the nursing service elderly satisfaction model. With the help of AMOS 22.0 software, we analyzed the degree of model fit and the behavioral relationships between the variables that affect the path. We found that the satisfaction of Chinese urban elderly individuals in nursing homes is at a moderate level, which is lower than the quality expected by elderly individuals. The customer satisfaction index model can be applied to assess satisfaction with nursing services. Furthermore, perceived quality and value have a significant impact on satisfaction.

## 1. Introduction

### 1.1. Background

Since the second half of the 20th century, the decline in fertility levels and the increase in life expectancy have become the main themes of world population development. The global population over the age of 60 is increasing at a rate of approximately 3% per year [1]. It is estimated that by 2050, the proportion of elderly individuals in all regions except Africa will be close to or greater than 25%, and the global ageing trend will be difficult to reverse [2]. The ageing of the population continues to deepen, with elderly individuals placing a huge burden on families and society. Driven by ageing, as well as the development of industrialization and urbanization, many urban elderly individuals in China are quicker than rural elderly individuals to change their concept of old-age care and enter nursing homes for care in their old age [3].

**Figure 1:**
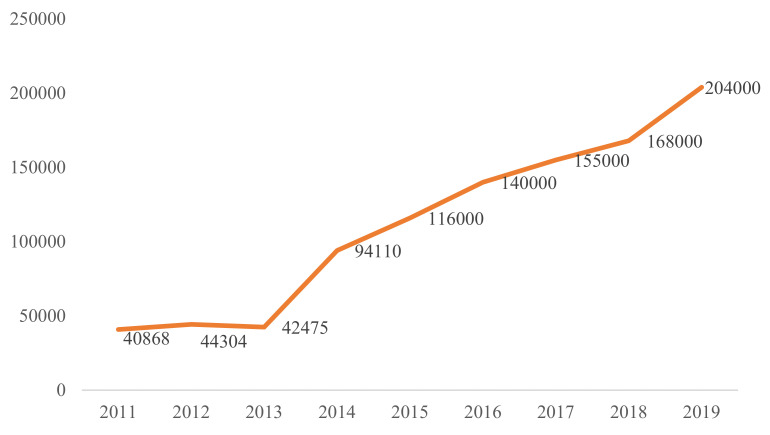
The growth in the number of nursing homes in China (2011–2019).

Since 2011, professional elderly nursing homes have played an increasingly important role in the development of the elderly care service industry [4]. Various types of elderly nursing homes in China have increased from 40,900 in 2011 to 204,000 in 2019 [5], and the elderly care service industry has rapidly developed (see Figure 1). However, the development of the nursing care service industry in China, like other developing countries, is relatively late, and the quality of service is uneven [6]. At present, nursing homes in China are in short supply. The elderly in China needs 8 million beds, but currently there are only 2.662 million [7], which implicates that there is a large gap between supply and demand. The supply of old-age service facilities is mainly in the form of private operation. There are various old-age services on the market, and the elderly can choose freely according to their personal needs and preferences. The prices of each nursing home are inconsistent, and there are also gaps in the quality of services corresponding to different prices. Take a nursing home in Jiangsu as an example. Its charging items include bed fees, service fees, meal fees, special service fees, and medical rehabilitation fees. The types of rooms provided range from double, quadruple, and quintuple rooms, and a monthly fee ranging from CNY 6800 to CNY 25000 [8] is established based on the ability of the elderly or children to pay. The high-quality development of nursing homes for elderly individuals is not only conducive to taking care of elderly individuals in their old age but also conducive to the stable development of an ageing society [9]. Therefore, at present, what is the quality of elderly care services in China, and what is the level of satisfaction of elderly individuals in nursing homes with the services of the nursing homes? In addition, starting on January 19, 2020, COVID-19 broke out in Wuhan and quickly spread to 34 provinces in China. Studies have found that elderly people with chronic diseases or compromised immune systems are at greater risk of infection [10]. After the outbreak, many nursing homes had cases of community infection, and they became high-risk places for virus infection. To reduce the risk of COVID-19 infection, many nursing homes have adopted closed management measures, such as declining visits by family members of the elderly [11]. During the outbreak, what has been the level of satisfaction of Chinese elderly individuals in nursing homes?

Regarding the satisfaction of urban elderly people with nursing services, some scholars have investigated elderly individuals in nursing homes in Kunming City [12] and Datong City [13]. The results show that elderly individuals are more satisfied in nursing services. The overall level of satisfaction is relatively high; however, some scholars [14] surveyed elderly individuals in 13 cities in Jiangsu Province and found that elderly residents are less satisfied with nursing services. In terms of the theoretical basis and methods of satisfaction evaluation, some academic circles take expectancy theory as the basis and analyze the gap between the service quality expected by elderly individuals and perceived service quality through confirmatory factor analysis and exploratory factor analysis to evaluate nursing service satisfaction evaluation. Some scholars have also used SERVQUAL [15] and the analytic hierarchy process (AHP) [16] models to set up more detailed service evaluation indicators to evaluate nursing service satisfaction. In recent years, some scholars have explored the use of the customer satisfaction model (American Customer Satisfaction Index (ACSI)) to evaluate satisfaction with elderly care services [17]. Zheng Shengqin [18] and others proposed that they can try to use the customer satisfaction model to study the nursing service satisfaction of urban elderly people. At the same time, when using the customer satisfaction model for evaluation, studies have found that the expectations, perceived quality, and perceived value of elderly individuals have a significant positive impact on their satisfaction and indirectly affect their loyalty through satisfaction. However, scholars such as Liu Wei [19] conducted a satisfaction evaluation of 16 elderly care institutions in Harbin City, finding that there is no direct relationship between expected quality and nursing service satisfaction.

Among the existing research conclusions, those on the degree of satisfaction of Chinese elderly people with nursing services are not consistent. Some scholars believe that elderly people are more satisfied with nursing services, while others believe that most elderly people are not satisfied with nursing services. At the same time, the academic community has just begun to try to use the customer satisfaction model to study satisfaction with elderly care services. Among the existing research conclusions, some scholars believe that expected quality can significantly affect the satisfaction of elderly people with nursing services. However, other scholars have found that there is no correlation between the quality expected by and the satisfaction of elderly people with nursing services, and the research conclusions are contradictory. Taking China as an example, this study tries to use the ACSI model to evaluate the satisfaction of urban elderly people with nursing services, finding deficiencies in elderly care services. This study provides a basis for decision-making for the development of the elderly care industry in China. At the same time, by studying the effects of factors such as perceived quality and perceived value, it provides support for improving customer satisfaction models. Based on the inconsistency between customer satisfaction theory and expectation inconsistency theory, this paper proposes hypotheses and constructs theoretical models, conducts online questionnaire surveys of elderly individuals in China, and uses structural equation modeling (SEM) to verify the research hypotheses and validate theoretical models.

### 1.2. Hypothesis Development

We established the theoretical basis of the article based on customer satisfaction theory and expectation inconsistency theory.

In 1960, Cardozo [20] conducted the first experimental study on customer satisfaction in the field of marketing and proposed that customer satisfaction would drive repurchase behavior. In 1989, scholars such as Sweden [21] proposed Swedish customer satisfaction theory on this basis. Fornell [22] and others believe that analyses of customer satisfaction also need to consider perceived quality and, on this basis, propose the ACSI model.

Expectation inconsistency theory holds that customers have an expectation of a product’s utility before purchasing the product and then compare their actual utility perception with their expectation after using the product. When the utility perception just reaches the expected level, it will produce a “completely consistent” mental state; exceeding the expected level will produce a “positive inconsistency” mental state; when it is below the expected level, a “negative inconsistent” mental state will occur. This consistent or inconsistent psychological state produced by the interaction between the expected level and the actual perception will directly affect the customer’s satisfaction with the product or service. Negative inconsistency will lead to customer dissatisfaction, positive inconsistency will lead to customer surprises beyond satisfaction, and complete agreement will result in customer satisfaction [23]. This theory believes that customer satisfaction is the result of customers comparing their expectations of a product before purchase with the actual experience after purchase. There is a non-linear relationship between different levels of customer satisfaction and customer loyalty, and the perceived value and expectations of products/services will affect customer satisfaction.

#### 1.2.1. Expected Quality

Expected quality means that customers will have expectations of quality based on past experience or external information before using products or services [24]. Expectation inconsistency theory holds that the expectations formed by customers before consumption are compared with their actual feelings of consumption and that the size and direction of the gaps produced determine whether customers are satisfied and the degree of satisfaction [25]. The expectation inconsistency model points out that the gap between the actual performance level of consumer products and the expectations before purchase will make customers have different “satisfying” responses. At the same time, Gronroos [26] believes that service quality refers to the difference between service expectations and perceived service performance. When the service expectation is higher than the perceived service performance, the service quality is poor; conversely, when the perceived service performance is higher than the service expectation, the service quality is better. Based on the above analyses, the following hypotheses (H1-H3) are proposed:

**Hypotheses** **1** **(H1).** *Expected quality is positively correlated with elderly satisfaction.*

**Hypotheses** **2** **(H2).** *Expected quality is positively correlated with perceived value.*

**Hypotheses** **3** **(H3).** *Expected quality is positively correlated with perceived quality.*

#### 1.2.2. Perceived Quality

Perceived quality is the subjective feelings of customers about the quality of the products or services they consume [27]. These feelings of experience will be one of the key research contents of the entire evaluation model. Wang Feiyan and Li Zuwu [28] believe that service is unique. Customer satisfaction with service-oriented enterprises is considered based on the entire service consumption process. Service (perceived) quality is the core factor that affects customer satisfaction and is the premise of customer satisfaction. Conducting empirical research, Zeithaml [29] concluded that service quality has a significant positive impact on cognitive value. When elderly people choose nursing homes, they have psychological expectations based on the information they have obtained. After living in nursing homes and actually experiencing the quality of their services, they will have a satisfaction evaluation of the services of the nursing homes. The higher the satisfaction level is, the easier it will be to re-use such services. Based on the above analyses, the following hypotheses (H4-H5) are proposed:

**Hypotheses** **4** **(H4).** *Perceived quality is positively correlated with elderly satisfaction.*

**Hypotheses** **5** **(H5).** *Perceived quality is positively correlated with perceived value.*

#### 1.2.3. Perceived Value

Perceived value is defined as the overall evaluation of product effectiveness based on customers’ total perceived costs and gains. In sociology, psychology, and organizational behavior, fairness is a concept that has received considerable attention; Oliver [30] and others have performed a series of studies. The results show that whether customers are satisfied with a product depends not only on their comparison between expectations and performance but also on whether customers believe that the transaction is fair and reasonable. When customers feels that the ratio of utility to investment is the same as that of the product provider, they will feel that the transaction was fair and will be satisfied. The higher the degree of fairness is, the more satisfied the customers are. Conversely, the lower the degree of fairness is, the more dissatisfied the customers are. After elderly individuals actually perceive nursing services, whether the perceived quality is worth the cost will produce satisfaction. Based on the above analysis, the following hypothesis is proposed:

**Hypotheses** **6** **(H6).** *Perceived value is positively correlated with elderly satisfaction.*

#### 1.2.4. Elderly Support

Customer loyalty (elderly support) refers to the possibility of customers repeating purchases and reserved prices [31]. Customer satisfaction (elderly satisfaction) is the level of a person’s psychological state, reflecting the basic attitude of a customer towards products or services, and it is the key to whether consumption can be repeated. In a study of the factors affecting nursing service satisfaction, Hua Zhaohong [32] found that elderly people’s satisfaction with nursing services was strongly correlated with their intention to use such services and that the elderly consumed the services again if they were satisfied. In this article, elderly support is the intention of elderly individuals, and elderly individuals will continue to support nursing services if they are satisfied with such services. Based on the above analysis, the following hypothesis is proposed:

**Hypotheses** **7** **(H7).** *Elderly satisfaction is positively correlated with elderly support.*

### 1.3. Model Construction

**Figure 2:**
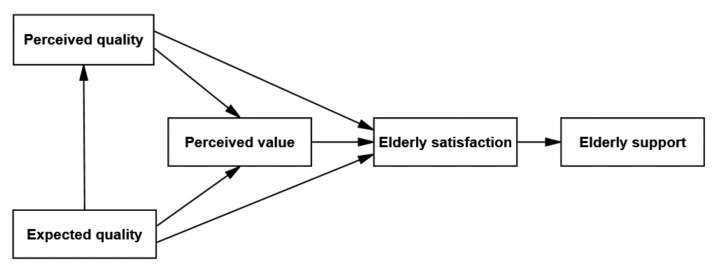
Nursing service elderly satisfaction model.

Based on the previous assumptions, we draw on the ACSI model to construct a nursing service elderly satisfaction model. Nursing service satisfaction is the cumulative evaluation by elderly individuals of the entire service process, and cumulative customer satisfaction can better predict customer loyalty. Therefore, we reduce the “customer complaint” latent variable in the ACSI model and adjust the “customer expectations, perceived quality, perceived value, customer complaints, and customer loyalty” in the original model into five latent variables: “expected quality, perceived quality, perceived value, elderly satisfaction, elderly support” with regard to nursing services. The constructed theoretical model is shown in Figure 2.

### 1.4. Variable Index

Elderly satisfaction and elderly support are the endogenous latent variables of this model, and expected quality, perceived quality and perceived value are its exogenous latent variables. Since the five latent variables, i.e., expected quality, perceived quality, perceived value, elderly satisfaction, and elderly support, cannot be directly observed, we set up 16 observed variables to measure them accordingly. Refer to Table 1 for the index system of the nursing service elderly satisfaction model.

#### 1.4.1. Endogenous Latent Variables: Elderly Satisfaction and Elderly Support

In this model, elderly satisfaction refers to the evaluation of the entire service by elderly individuals who have been admitted to a nursing home after consuming the services of the nursing home. It is the overall evaluation of elderly individuals’ overall consumption experience of the entire nursing service process. Elderly satisfaction is measured based on four dimensions: expected quality, perceived quality, perceived value, and elderly support. Elderly support is the choice after the actual experience of nursing services, based on satisfaction with the behavioral decisions elderly individuals will make. Elderly support is manifested by the willingness to buy again and the willingness to recommend to others.

#### 1.4.2. Exogenous Latent Variables: Expected Quality, Perceived Quality, and Perceived Value

Expected quality refers to elderly individuals’ estimate of the quality of nursing homes before choosing nursing homes. Perceived quality is occupants’ overall evaluation of the services they consume in the nursing home, and it is the multidimensional perception of the occupants as customers. Perceived value represents the perceived level of service quality relative to price. Based on the SERVQUAL scale jointly developed by Parasuraman, Zeithaml, and Berry (PZB) in 1988, we measure elderly satisfaction based on five dimensions of tangibility, reliability, responsiveness, safety, and empathy with the establishment of expected quality and perceived quality.

## 2. Materials and Methods

### 2.1. Data Source

Based on the hypotheses, we designed a questionnaire. The questionnaire mainly consists of two parts. The first part concerns the demographic characteristics of the respondents, including their gender, age, educational level, economic level, etc. The second part incorporates the SERVQUAL method to establish observable indicators based on five aspects: tangibility, responsiveness, reliability, security, and empathy. We use a 5-point Likert scale for subjective evaluation and assign 1, 2, 3, 4, and 5 points for very dissatisfied, dissatisfied, neither dissatisfied nor satisfied, satisfied, and very satisfied, respectively, so that elderly individuals can more clearly express their satisfaction.

The survey subjects were urban elderly people over 60 years old across China who consumed nursing services. We used an online questionnaire survey system (Questionnaire Star: http://www.wjx.cn 15 April 2021) and the snowball method to invite samples to complete the questionnaire online. We first selected 10 elderly people who had consumed nursing home services and asked them to complete the questionnaire online. After they did so, they were asked to provide other subjects belonging to the target population under study. We used this method to obtain a large number of samples. To control for questionnaire quality, the same IP address could complete the questionnaire only once. Private information, such as individuals’ names, was not involved in the questionnaire, and sensitive language was avoided.

**Figure 3:**
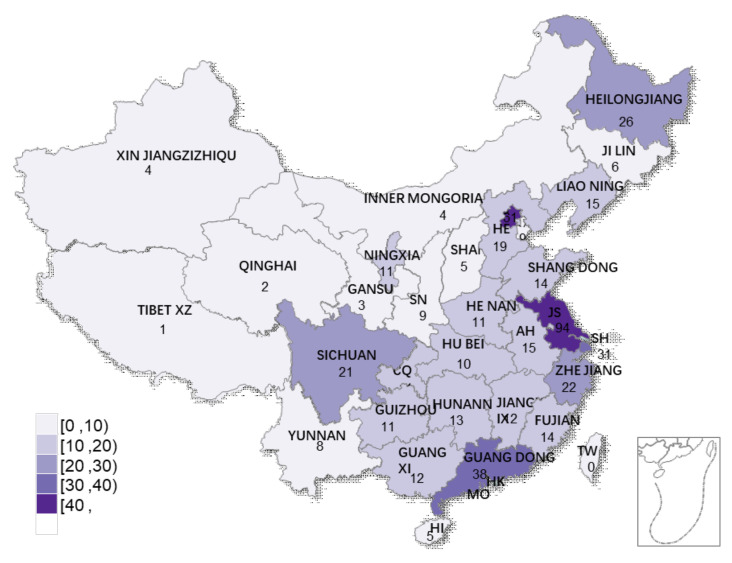
Survey sample distribution.

The survey started on March 22, 2021 and ended on April 15, 2021. A total of 533 questionnaires were collected. After screening, 517 valid questionnaires were obtained, for an effective response rate of 96.99%. The population distribution of China is 39.93% in the eastern region, 25.83% in the central region, 27.12% in the western region, and 6.98% in the northeast region [33]. The survey samples involved 307 samples from Eastern China, 66 samples from Central China, 97 samples from Western China, and 47 samples from Northeast China, which conformed to the law of China’s population distribution. The data are representative. The sample area is shown in Figure 3.

### 2.2. Methods

First, we used SPSS 22.0 statistical software (SPSS Inc., Chicago, IL, USA) to test whether the latent variables were reasonable and valid based on the Cronbach’s α reliability coefficient and the Kaiser–Meyer–Olkin (KMO) value. At the same time, we performed descriptive statistical analysis of the demographic characteristics of the sample. Second, through SEM, which was carried out to test the nursing service elderly satisfaction model, we assumed that there was a causal relationship between variables and that latent variables could be measured by explicit variables. By verifying the covariance between the variables, the linear regression coefficients between the variables were estimated. Finally, we used AMOS 22.0 software (SPSS Inc., Chicago, IL, USA) to obtain the ratio of the X2 and the degrees of freedom ratios (X2/df), the Tucker–Lewis index (TLI), the incremental fit index (IFI) and other indexes and tested the model fit and the behavioral relationships between the variables that affect the path. The specific expressions are as follows:

(1) $$X=\Lambda_{x}\xi +\theta$$

(2) $$Y=\Lambda_{y}\eta +\delta$$

(3) $$\eta ={Β}_{\eta }+\Gamma_{\xi }+\varepsilon$$

Equations (1) and (2) are measurement equations that represent the relationship between latent variables and explicit variables. *X* is a vector composed of exogenous explicit variables, *Y* is a vector composed of endogenous explicit variables, *Λy*, *Λx* is the relationship matrix between latent variables and explicit variables, *ξ* is a vector composed of exogenous latent variables, *η* is a vector composed of endogenous latent variables, and *δ* and *θ* are error terms.

(4) $$\left\{\begin{array}{c} \eta_{1}\\ \eta_{2}\\ \eta_{3}\\ \eta_{4}\\ \eta_{5} \end{array}\right\}=\{\begin{array}{cccccc} 0 & 0 & 0 & 0 & 0 & 0\\ \beta_{21} & 0 & 0 & 0 & 0 & 0\\ \beta_{31} & \beta_{32} & 0 & 0 & 0 & 0\\ 0 & \beta_{42} & \beta_{43} & 0 & 0 & 0\\ 0 & 0 & 0 & \beta_{54} & 0 & 0\\ 0 & 0 & 0 & \beta_{64} & \beta_{65} & 0 \end{array}\}\left\{\begin{array}{c} \eta_{1}\\ \eta_{2}\\ \eta_{3}\\ \eta_{4}\\ \eta_{5} \end{array}\right\}+\xi \left\{\begin{array}{l} \gamma_{1}\\ 0\\ \gamma_{3}\\ 0\\ 0 \end{array}\right\}+\left\{\begin{array}{c} \xi_{1}\\ \xi_{2}\\ \xi_{3}\\ \xi_{4}\\ \xi_{5} \end{array}\right\}$$

Equation (4) corresponds to Equation (3), and *β* and *γ* are the path coefficients of the equation. Equation (3) is the structural equation, *η* is the endogenous latent variable (elderly satisfaction *η*_1_, elderly support *η*_2_), *ξ* is the exogenous latent variable (expected quality *ξ*_1_, perceived quality *ξ*_2_, perceived value *ξ*_3_), and В is the relationship between endogenous latent variables and the influence relationship between exogenous latent variables and endogenous latent variables; *ε* is the residual term of the endogenous latent variable.

## 3. Results

### 3.1. Variable Descriptive Statistics

#### 3.1.1. Sample Population Attributes

Among the survey samples, Table 2 shows the basic demographic characteristics of the survey samples. In terms of gender, the proportion of women was 56.1%, and the proportion of men was 43.9%. In terms of age, those aged 60–65 years old accounted for 24.8%, those aged 66–70 years old accounted for 30.6%, those aged 71–75 years old accounted for 17.4%, those aged 76–80 years old accounted for 20.3%, and those aged above 80 years old accounted for 7.0%. In terms of educational level, the proportion of uneducated individuals in the sample was 22.6%, 20.5% had an educational level of elementary school, those with a junior high school educational level accounted for 16.2%, 15.9% of the sample had a high school education, the proportion with an undergraduate/bachelor’s degree was 19.5%, and the proportion with a graduate degree and above was 5.2%. According to the survey results, more than half of the people had an annual income of less than CNY 80,000.

#### 3.1.2. Descriptive Statistics of the Latent Variables

We used SPSS 22.0 statistical software to make necessary corrections to the survey data or to eliminate some abnormal data from the questionnaire to obtain the value of the latent variables in the nursing service elderly satisfaction model. As shown in Table 3, the standard deviation of expected quality is 1.29, indicating that due to individual differences, elderly individuals have diverse expectations of the quality of nursing services. The average t score of elderly support is 2.92, which is at a low level, and the interaction with other variables needs further research.

### 3.2. Reliability and Validity Tests

SPSS 22.0 was used to analyze the validity of the questionnaire. As shown in Table 4, the statistical value of the KMO test of validity is 0.834, which is greater than the standard of 0.7; the *p* value of Bartlett’s test of sphericity is 0.000, which shows that the reliability of the questionnaire is strong and that its internal consistency is acceptable.

At the same time, the reliability test of each part of the questionnaire scale shows in Table 5 that the expected quality α is 0.943, the perceived quality α is 0.901, the perceived value α is 0.855, the elderly satisfaction α is 0.670, and the elderly support α is 0.813. The reliability coefficients of all variables meet the requirements, indicating that the latent variables of this questionnaire have good internal reliability.

### 3.3. Model Checking

#### 3.3.1. Confirmatory Factor Analysis

AMOS 22.0 was used to perform confirmatory factor analysis of the nursing service elderly satisfaction model, and the observed variables corresponded to the six latent variables. The results show that the factor loading of each observed variable is greater than 0.5. The loading coefficients of the 16 observed variables are all greater than 0.5, indicating that the observed variables have good explanatory power (Table 6).

#### 3.3.2. Model Fit Test

To test the degree of fit of the elderly care service satisfaction model (Table 7), the commonly used indicators are the X2/df, TLI, IFI, comparative fit index (CFI), goodness-of-fit index (GFI), parsimonious goodness-of-fit index (PGFI), and parsimonious normed fit index (PNFI). It is generally considered that when a model’s X2/df < 2, its TLI, IFI, CFI, GFI, and non-normed fit index (NNFI) ≥ 0.90, and its PGFI and PNFI ≥ 0.50, the model fits well. Table 7 shows the fit indexes of the nursing service satisfaction model. The test results of all indicators of the nursing service elderly satisfaction model meet the model fit standards; thus, the nursing service elderly satisfaction model has a good degree of model fit.

#### 3.3.3. Path Coefficients

The path coefficient test of the elderly care service satisfaction model was carried out, and the results are shown in Table 8. The test found that expected quality has no significant impact on elderly satisfaction (H1); additionally, expected quality has no significant impact on perceived value (H2) or perceived quality (H3). The results of the analysis of the path coefficients of the nursing service elderly satisfaction model ultimately show that there are four significant paths.

#### 3.3.4. Model Running Results

**Figure 4:**
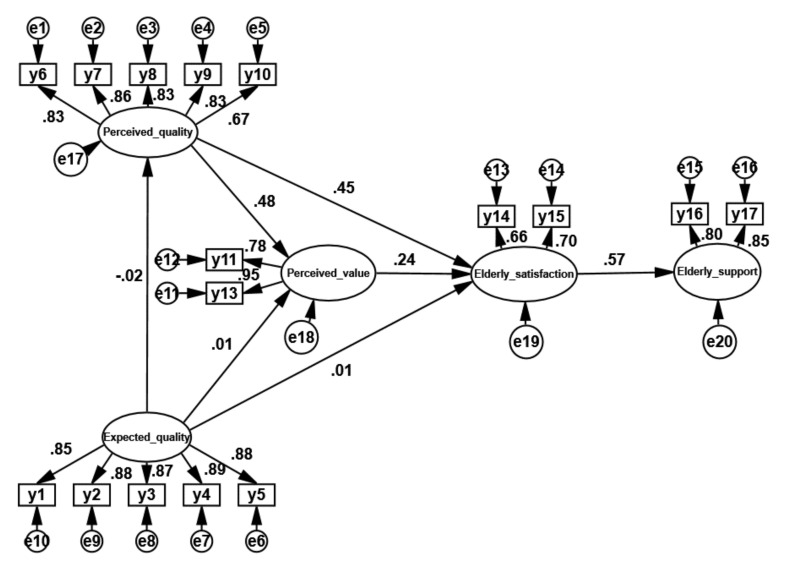
Path coefficients of the nursing service elderly satisfaction model.

SEM was used to verify the constructed nursing service satisfaction model, and SPSS 22.0 and AMOS 22.0 software were used to analyze the data of the nursing service satisfaction survey questionnaire. Finally, the relationships between the various latent variables of the nursing service satisfaction model were calculated. The path coefficients are shown in Figure 4.

**Figure 5:**
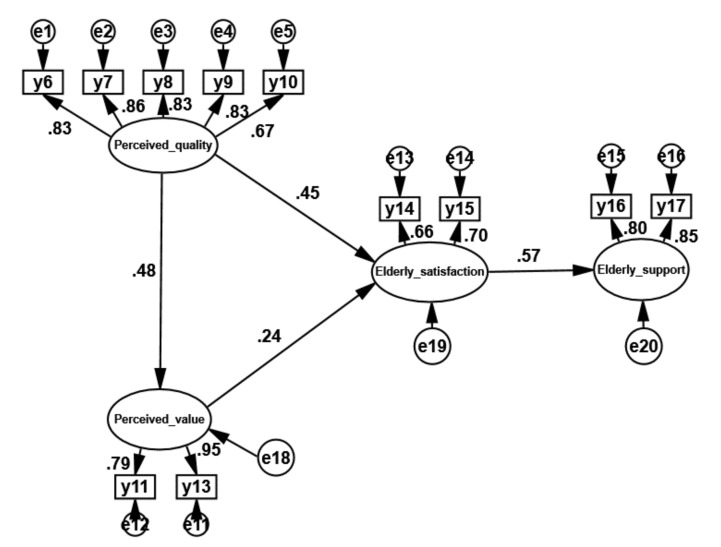
Modified path coefficients of the elderly satisfaction model of nursing services.

Figure 4 shows that the coefficient of the path from perceived quality to perceived value is 0.48 and that the coefficient of the path from perceived quality to elderly satisfaction is 0.45. The largest path coefficient in the whole model is the coefficient of the path from elderly satisfaction to elderly support, which is 0.57. The coefficient of the path from perceived value to elderly satisfaction is 0.24. The coefficient of the path from expected quality to perceived quality is −0.02, the coefficient of the path from expected quality to perceived value is 0.01, and the coefficient of the path from expected quality to elderly satisfaction is 0.01. Combined with the path significance test in Table 7, the three paths from expected quality to perceived quality, perceived value, and elderly satisfaction are not significant. Therefore, the model is modified to obtain Figure 5.

After removing the latent variable of expected quality, a revised model is obtained. The model includes four latent variables: perceived quality, perceived value, elderly satisfaction, and elderly support. AMOS 22.0 was used to test the model fit and to conduct the significance test of the related latent variables. The results are shown in Table 9 and Table 10.

## 4. Discussion

As the main way of providing socialized elderly care services, nursing home care services can provide comprehensive care for elderly individuals [34]. Increasingly, nursing homes have become the main old-age care provider for meeting the diversified service needs of elderly individuals and improving their quality of life. Based on the ACSI model, this paper constructs a nursing service elderly satisfaction model. The main findings of this research are as follows:

1. The elderly individuals in the sample were generally satisfied with the nursing services in nursing homes.

The analysis results show that during the epidemic, elderly individuals’ satisfaction score for nursing services was 3.08 ± 0.74 points (out of 5) and that there is a certain gap compared with the expected quality (3.33 ± 1.29). These results show that in China, which is still in the developing stage, the country’s nursing services still have considerable room for improvement. In recent years, although China’s elderly care service industry has developed rapidly, there is still insufficient supply [35]. With the gradual improvement of material living standards, the consumer demand for nursing services for urban elderly individuals is gradually escalating, and such individuals have higher expectations of nursing services [36]. The elderly care service industry, which is still transitioning from supply scale expansion to high-quality supply, is still unable to meet the higher-level elderly care needs of elderly people [37]. Elderly people are more sensitive to higher-level nursing services and have a lower level of satisfaction with nursing services [38]. At the same time, special management measures such as reduced visits during the epidemic may have had a certain negative impact on elderly satisfaction.

2. Expected quality has no significant impact on perceived quality, perceived value, or elderly satisfaction.

Lei Jie [39] found that public expectations are a non-negligible factor affecting public satisfaction. For every one-unit reduction in public expectations, public satisfaction increases by 0.07 units. Zhu Hanxiao [40] conducted a satisfaction evaluation of a medical care integrated nursing home and found that the expectations, perceived quality, and perceived value of elderly individuals all have varying degrees of impact on their satisfaction. However, this article found that the three paths from expected quality to elderly satisfaction, perceived value and perceived quality are not significant, which is different from the established cognition. This article believes that elderly people’s demand for nursing services is hierarchical, diverse, and highly flexible [41]. Due to differences in demographic characteristics such as age, gender, place of residence, and income, elderly individuals have different needs for nursing services [42], and there are also differences in their expectations of nursing services [43]. Most elderly people do not have much long-term experience with nursing services; thus, their expectations of nursing services are not well expressed [44]. For example, Shanghai SY Nursing Home has introduced an internationally leading continuous care retirement community elderly care model and is equipped with professional rehabilitation hospitals and professional elderly care equipment to meet the needs of high-income elderly. Regarding the supply of nursing services in China, which is still in the developing stage, it is still unable to meet the needs of the elderly individuals. There is much room for the development of the elderly care industry.

3. Perceived quality significantly positively affects perceived value and elderly satisfaction.

Perceived quality is measured by five observed variables: whether the nursing service items are rich, the attitude of the nursing staff, the suitability of the environment, the reliability of the nursing staff, and consistency before and after admission to the nursing home. Regarding the effect of perceived quality on elderly satisfaction, the path coefficient is 0.45, which shows that elderly individuals’ evaluation of their satisfaction with nursing services is affected by the five aspects above. In the process of providing nursing care, nursing homes can effectively improve customer satisfaction if the above-mentioned aspects of service quality are improved. On the contrary, it will reduce the satisfaction of the elderly. For example, Changsha AX Nursing Home has different promises before and after the elderly move in, resulting in very low satisfaction for the elderly. The influence of perceived quality on perceived value has a path coefficient of 0.48. When customers measure whether the cost is worthwhile, they comprehensively consider the actual perceived quality to evaluate their satisfaction with nursing services.

Most elderly individuals are cost-effective consumers. The lower the cost-effectiveness and the higher the dissatisfaction of elderly individuals are, the lower the quality and profit of nursing services in nursing homes [45]. Therefore, nursing homes should strengthen the contents of their services, service facilities and service teams to ensure the reasonable pricing of nursing services, which can improve the cost-effectiveness of such services.

## 5. Conclusions

Based on customer satisfaction theory, we constructed a nursing service elderly satisfaction model and used SEM to verify the research hypotheses and validate the theoretical model. Studies have shown that the overall satisfaction with the nursing services provided by nursing homes for elderly individuals in Chinese cities is average, which is lower than the expectations of these individuals. The research also found that expected quality has no significant impact on elderly satisfaction, while perceived quality significantly positively affects perceived value and elderly satisfaction. This research is limited to model testing and fails to discuss in detail the influencing factors of expected quality. In reality, there may be more influencing factors related to expected quality that affect nursing service satisfaction considering the rapid change of COVID-19 [46,47,48,49,50]. In addition, due to differences in the level of economic and social development, the supply of nursing services in Eastern, Central, and Western China may have certain differences. We do not reflect the diversity of nursing service supply in the different regions of China.

## Figures and Tables

**Table 1:** Nursing service elderly satisfaction index system.

Variable	Number	Measurement
Expected quality	5	Nursing service items
		Attitudes of nursing staff
		Environmental suitability
		Nursing staff reliability
		Nursing home trustworthiness
Perceived quality	5	Nursing service items
		Attitudes of nursing staff
		Environmental suitability
		Nursing staff reliability
		Nursing home trustworthiness
Perceived value	2	See price for a given quality
		See quality at a given price
Elderly satisfaction	2	Elderly satisfaction compared with ideals Elderly satisfaction compared with expectations
Elderly support	2	Whether to accept elderly care services again
		Whether to recommend to others

**Table 2:** Descriptive statistics of the sample population attributes.

Variable	Content Composition	Percentage (%)
Gender	Female	56.10%
	Male	43.90%
Age	60–65 years old	24.80%
	66–70 years old	30.60%
	71–75 years old	17.40%
	76–80 years old	20.30%
	Over 80 years old	7.00%
Educational level	Uneducated	22.60%
	Primary school	20.50%
	Junior high school	16.20%
	High school	15.90%
	Undergraduate/bachelor’s degree	19.50%
	Graduate school and above	5.20%
Annual income	Below 20,000	22.20%
	20,000–30,000	28.00%
	30,000–50,000	15.70%
	50,000–80,000	13.30%
	80,000–100,000	5.40%
	100,000–150,000	6.60%
	More than 150,000	8.70%

**Table 3:** Descriptive statistics of the latent variables.

Variable	Average	Median	Standard Deviation
Expected quality	3.33	3.80	1.29
Perceived quality	3.74	4.00	0.96
Perceived value	3.07	3.00	0.83
Elderly satisfaction	3.08	3.00	0.74
Elderly support	2.92	3.00	0.79

**Table 4:** KMO and Bartlett’s tests.

Kaiser–Meyer–Olkin Measure of Sampling Adequacy		0.834
Bartlett’s test of sphericity	Approximate chi-square	5951.300
	df	210
	Significance	0.000
Cronbach’s α		0.824

**Table 5:** Reliability testing.

Variable	Cronbach’s α
Expected quality	0.943
Perceived quality	0.901
Perceived value	0.855
Elderly satisfaction	0.670
Elderly support	0.813

**Table 6:** Factor loading detection.

Variable	Measurement Indicators	Factor Loading
Expected quality	Y1	0.848
	Y2	0.885
	Y3	0.873
	Y4	0.893
	Y5	0.881
Perceived quality	Y6	0.826
	Y7	0.858
	Y8	0.832
	Y9	0.834
	Y10	0.674
Perceived value	Y11	0.784
	Y12	0.953
Elderly satisfaction	Y13	0.665
	Y14	0.702
Elderly support	Y15	0.804
	Y16	0.853

**Table 7:** Model fit test.

Compatibility Index	X2/df	CFI	GFI	NFI	PGFI	PNFI	RMSEA	SRMR
Adaptation standard	<3	>0.90	>0.90	>0.90	>0.50	>0.50	<0.06	<0.08
Test result	1.610	0.988	0.963	0.970	0.687	0.783	0.034	0.046

**Table 8:** Significance test of the path coefficients.

No.	Dimensions	Estimate	*p*	Test Result
H1	Elderly satisfaction ← Expected quality	0.003	0.872	Rejected
H2	Perceived value ← Expected quality	0.010	0.735	Rejected
H3	Perceived quality ← Expected quality	−0.019	0.603	Rejected
H4	Elderly satisfaction ← Perceived quality	0.262	***	Accepted
H5	Perceived value ← Perceived quality	0.433	***	Accepted
H6	Elderly satisfaction ← Perceived value	0.155	***	Accepted
H7	Elderly support ← Elderly satisfaction	0.706	***	Accepted

Note: *** *p* < 0.01.

**Table 9:** Modified model fit test.

Compatibility Index	X2/df	CFI	GFI	NFI	PGFI	PNFI	RMSEA	SRMR
Adaptation standard	<3	>0.90	>0.90	>0.90	>0.50	>0.50	<0.06	<0.08
Test result	2.325	0.981	0.968	0.968	0.587	0.704	0.051	0.048

**Table 10:** Significance test of the latent variables.

	Estimate	S.E.	C.R.	*p*
Elderly satisfaction ← Perceived quality	0.433	0.041	10.552	***
Perceived value ← Perceived quality	0.156	0.039	3.958	***
Elderly satisfaction ← Perceived value	0.262	0.037	7.050	***
Elderly support ← Elderly satisfaction	0.707	0.089	7.977	***

Note: *** *p* < 0.01.

## Data Availability

Not applicable.
